# All-optical ultrasound catheter for rapid B-mode oesophageal imaging

**DOI:** 10.1364/BOE.494878

**Published:** 2023-07-11

**Authors:** India Lewis-Thompson, Edward Z. Zhang, Paul C. Beard, Adrien E. Desjardins, Richard J. Colchester

**Affiliations:** 1 Department of Medical Physics and Biomedical Engineering, University College London, Gower Street, London, WC1E 6BT, UK; 2 Wellcome/EPSRC Centre for Interventional and Surgical Sciences, University College London, Charles Bell House, Foley Street, London, W1W 7TY, UK

## Abstract

All-optical ultrasound (OpUS) is an imaging paradigm that uses light to both generate and receive ultrasound, and has progressed from benchtop to *in vivo* studies in recent years, demonstrating promise for minimally invasive surgical applications. In this work, we present a rapid pullback imaging catheter for side-viewing B-mode ultrasound imaging within the upper gastrointestinal tract. The device comprised an ultrasound transmitter configured to generate ultrasound laterally from the catheter and a plano-concave microresonator for ultrasound reception. This imaging probe was capable of generating ultrasound pressures in excess of 1 MPa with corresponding −6 dB bandwidths > 20 MHz. This enabled imaging resolutions as low as 45 µm and 120 µm in the axial and lateral extent respectively, with a corresponding signal-to-noise ratio (SNR) of 42 dB. To demonstrate the potential of the device for clinical imaging, an *ex vivo* swine oesophagus was imaged using the working channel of a mock endoscope for device delivery. The full thickness of the oesophagus was resolved and several tissue layers were present in the resulting ultrasound images. This work demonstrates the promise for OpUS to provide rapid diagnostics and guidance alongside conventional endoscopy.

## 1. Introduction

Ultrasound imaging can provide detailed visualisation of tissue for guidance and diagnostics during minimally invasive surgery. Conventionally, piezoelectric transducers are used, which use electricity to generate and receive the ultrasound [1,2]. However, it can be challenging to achieve adequate reception sensitivity and wideband transmission using miniaturised piezoelectric components [3]. Recently, an alternative technique known as all-optical ultrasound (OpUS) has shown promise for these highly miniaturised applications.

With OpUS, light is used to both generate and receive the ultrasound signal. Ultrasound is generated via absorption of pulsed or modulated light within a coating material. This leads to a temperature rise and corresponding pressure change via the photoacoustic effect which propagates as an ultrasound wave [4]. Ultrasound reflections from tissue are subsequently received using optical interferometry [5,6]. This technique has advantages in terms of ease of miniaturisation through the use of optical fibres, high sensitivity, broad ultrasound bandwidth and immunity to electromagnetic interference [7,8]. Another promising feature of OpUS is the potential to integrate other optical modalities, such as photoacoustic imaging [9] or laser ablation [10], via additional optical fibres [7] or wavelength-selective coatings [11].

A majority of the devices demonstrated to date have utilised a forward-viewing configuration for synthetic aperture imaging in both two- [3,12–15] and three- [7,16] dimensions. Additionally, there have been a small number of studies in which *in vivo* tissue imaging was performed with devices suitable for minimally invasive imaging [17,18]. However, in many clinical contexts, it is desirable to have a side-view from the device to provide the operator with a view of the specific vessel wall, such as coronary arteries [19,20], branches of the bronchial tree [21], or the gastrointestinal tract [22]. Previous examples using laterally-viewing OpUS include rotational imaging of a swine carotid artery [8] and B-mode imaging of a swine aorta using a single optical fibre device [23]. However, with these previous cases, no housing was used and the devices would require modification to be used in a preclinical context.

In this work we overcome this limitation, designing an OpUS device with an ultrasonically transparent sheath and a pullback system to provide a sub-second image acquisition. The device was designed for application in the diagnosis of Barrett’s Oesophagus, which is the only recognised precursor of oesphageal adenocarcinoma [24,25]. In this context, OpUS could be used to provide information which is complementary to the optical endoscope imaging, without disturbing the current workflow. For example, the diagnosis and staging of Barrett’s Oesophagus is currently carried out via histopathology of biopsies collected under endoscopic guidance [25]. However, this methodology is at significant risk of sampling error; the likelihood of positive identification of Barrett’s Oesophagus is shown to correlate with the number of biopsies acquired [26,27]. The addition of OpUS imaging through this working channel might be used to provide a ’virtual biopsy’ [13,28] of the current stage of the disorder in real-time, as well as providing potential guidance for biopsy.

In this study, we designed an OpUS imaging probe, with lateral dimensions compatible with standard-of-care clinical endoscope working channels. The probe was housed within an ultrasound transparent sheath and was capable of providing 2 D B-mode ultrasound images with sub-second acquisition times. A resolution phantom was used to assess the probe and tissue imaging was demonstrated on an *ex vivo* swine oesophagus through a mock endoscope. The images acquired demonstrate the potential for this technology to provide complementary information to standard endoscope images.

## 2. Materials and methods

### 2.1 OpUS imaging system

For this study, an OpUS imaging system was developed which comprised two elements: an OpUS probe and an OpUS console. Each comprised several components, as detailed in the following sections.

#### 2.1.1 OpUS probe

##### OpUS Transmitter

**Fig. 1.**
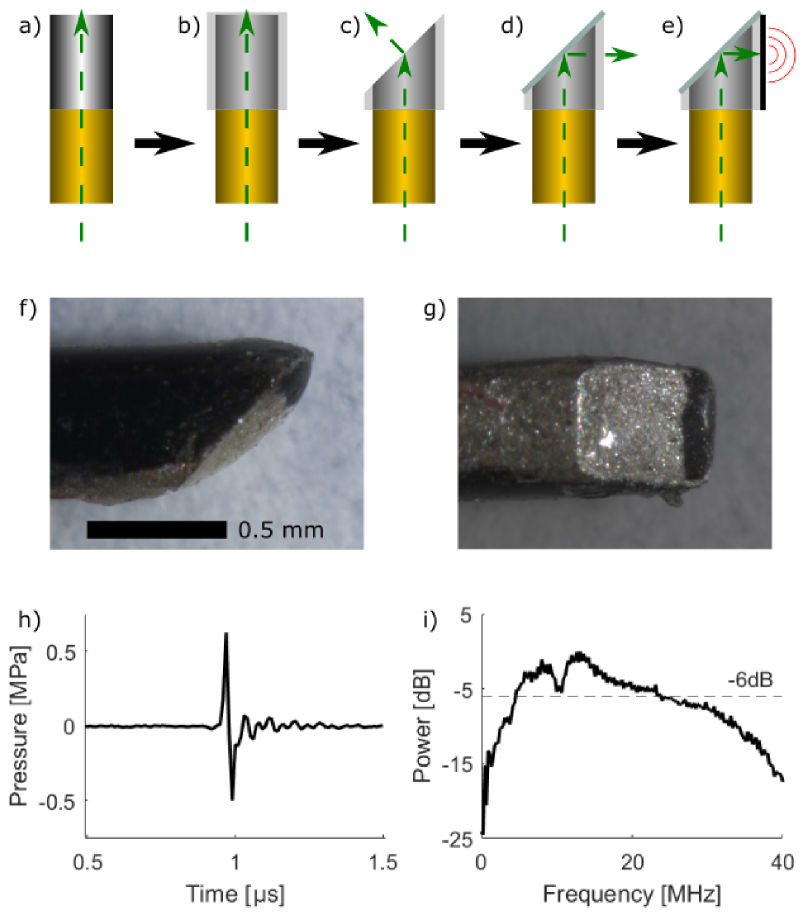
a) - e) OpUS transmitter fabrication process; a) stripped and cleaved optical fibre, b) optical fibre inserted in square glass capillary, c) optical fibre and capillary polished to a 45 °angle, d) silver mirror applied to polished surface, e) OpUS-generating composite coating applied to capillary surface. f) - g) Stereo-microscope images of lateral transmitter tip showing polished 45 °surface from f) the side-view (with scale bar) and g) the bottom view. h) Transmitted ultrasound time-series measured at 1.5 mm. i) Corresponding ultrasound power spectrum.

Side-viewing optical ultrasound transmitters which comprised an optical fibre and a square glass capillary were fabricated using a multi-step process (Fig. 1(a) - (e)). Firstly, SMA connectorized silica core/silica cladding optical fibre pigtails with a 200 µm core diameter and polyimide buffer coating (FG 200 LEP, Thorlabs, UK) were prepared by stripping the buffer coating from the last 10 mm of the optical fibre (Fig. 1(a)). Subsequently, square glass capillaries (OD: 0.5 x 0.5 mm, wall thickness: 0.1 mm, Vitrocom Hollow Square 8250−100 , CM Scientific, Germany) were cut to length (*ca.* 1 cm) using a tungsten blade. The stripped tip of the optical fibre was inserted into the square capillary such that their distal tips were aligned (Fig. 1(b)). The remaining space inside the capillary was filled with an optical epoxy (Norland Optical Adhesive 1665 , Edmund Optics, USA), which was cured to fix the optical fibre in place. Subsequently, the tip of the fibre, complete with capillary casing, was polished to 45 ° using a fibre polishing system (KrellTech, NJ, USA) (Fig. 1(c)). After polishing, silver mirror paint (186-3600, RS Pro, UK) was manually applied with a brush to the polished surface and left to dry for *ca.* 12 hours (Fig. 1(d)).

To fabricate the ultrasound generating coating, a method previously described was used [14]. Briefly, reduced graphene oxide (rGO) was dissolved in xylene and deposited on the capillary surface opposite the silver mirror and left to dry for *ca.* 12 hours (Fig. 1(e)). Subsequently, an overcoat of polydimethylsiloxane (PDMS)(MED- 1000 , Polymer Systems Technology, UK) thinned with xylene was applied to the rGO surface to create a bilayer composite.

The generated ultrasound was characterised by coupling the transmitter to the OpUS console. The excitation laser had a pulse energy of 20 µJ, corresponding to an incident fluence of 15.9 mJ/cm^2^ on the composite coating. The generated ultrasound was measured at a distance of 1.5 mm from the coating using a 200 µm needle hydrophone (Precision Acoustics, UK) with a calibrated range 1−30 MHz. The acquired time-series were Fourier transformed and the hydrophone calibration was applied to obtain the ultrasound bandwidth. The generated ultrasound peak-to-peak pressure level was measured as 1.12 MPa (Fig. 1(h)) at 1.5 mm from the coated capillary face with a corresponding −6 dB bandwidth of 22 MHz (Fig. 1(i)).

##### OpUS Receiver

The ultrasound receiver was fabricated using single-mode optical fibres with core/cladding diameters of 8/125 µm (SMF- 28 , Thorlabs, UK). A plano-concave microresonator was fabricated at the distal end by dip coating into an optically transparent polymer as described previously [16]. A dieletric mirror was applied to the fibre end prior to the polymer coating, with a second mirror deposited on the outer surface of the polymer. The mirror reflectivities were nominally 98 % in the range of 1500−1600 nm. Finally, the plano-concave microresonator was coated in a protective layer (thickness *ca.* 5 µm) of parylene C.

##### OpUS Probe Housing

**Fig. 2.**
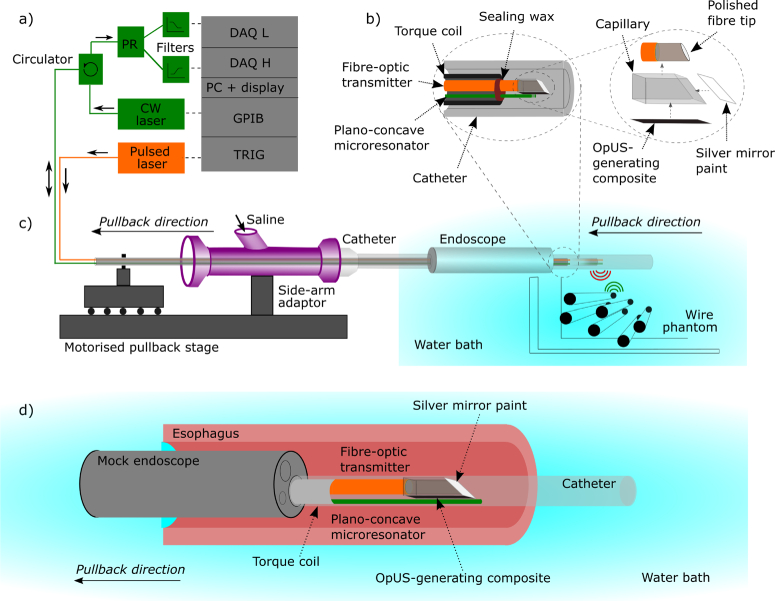
System for all-optical lateral ultrasound imaging through fast pullback. a) Imaging console comprising pulsed excitation light delivered to the transmission fibre and CW light delivered from a wavelength-tuneable laser to the receiving fibre. b) Cutaway schematic of the fabricated side-viewing OpUS probe comprising the plano-concave microresonator and side-viewing optical ultrasound transmitter. c) Schematic of the side-viewing optical ultrasound transducer. Ultrasound transmitting fibre (orange) and omnidirectional receiver (green) inside the probe, including OpUS probe submerged in the saline water bath and directed at the wire phantom imaging target. d) Schematic of lateral probe encased in an ultrasonically-transparent sheath inside the working channel of a mock endoscope inserted into an oesophagus.

To facilitate medical translation, the probe was incorporated within a medical grade catheter. Here, an ultrasonically transparent sheath was adapted from an endobronchial guide sheath (K 201 EBUS Sheath, Olympus, US). Within the probe, the ultrasound transmitter and receiver were held together inside a protective torque coil (ID: 0.8 mm, OD: 1.2 mm) (Fig. 2(b)). The distal tips of the transmitter and receiver were aligned longitudinally such that the receiving element extended *ca.* 1 mm beyond the transmitter. The two fibres were fixed in place using sealing wax and heat shrink tubing (Fig. 2(b)). The probe was then inserted into an ultrasonically-transparent sheath (ID: 1.4 mm, OD: 1.6 mm) (Fig. 2(b,c)) via a Y-piece connector (Fig. 2(c)) which allowed flushing with water during experiments. A mock endoscope was fabricated out of steel hypotubing using the same diameters as standard clinical gastrointestinal endoscopes: a 5 mm outer diameter with a 2 mm working channel [29]. The OpUS catheter was inserted through the working channel of the mock endoscope for imaging (Fig. 2(c)).

#### 2.1.2 Console

The console comprised optoelectronic components to both deliver pulsed excitation light to the OpUS transmitter and interrogate the OpUS receiver (Fig. 2(a)). For ultrasound generation, pulsed excitation light with a wavelength of 1064 nm, a pulse width of 2 ns, and a pulse energy of 20 µJ was delivered into the ultrasound transmission fibre from a Q-switched Nd:YAG laser (SPOT- 10 - 500 - 1064 , Elforlight, UK). The ultrasound receiving fibre was interrogated with continuous-wave light delivered by a tuneable laser (Tunics T 100 S-HP CL, Yenista Optics, France, tuning range: 1500−1630 nm, power: 4.5 mW). The continuous wave laser was connected to the hydrophone via a circulator (Fig. 2(a)). This allowed the reflection from the hydrophone to be monitored using a photoreceiver. The photoreceiver split the signal into a low frequency component ( <50 kHz) and high frequency component ( >500 kHz) (Fig. 2(a)). The low-frequency component was digitised at 16 bits with a sample rate of 1 MS/s and was used to record the Fabry-Pérot (FP) transfer function and to track the optimum bias point of the interferometer transfer function, as outlined previously [6]. The high-frequency component of the ultrasound signals were digitised at 14 bits with a sample rate of 100 MS/s. This was the ultrasound component originating from variations in the reflectivity of the FP cavity produced by impinging ultrasound waves.

### 2.2 OpUS imaging

#### 2.2.1 Data acquisition

The proximal end of the OpUS probe was mounted on a motorised translation stage (DDSM 100 , Thorlabs, UK, maximum speed: 500 mm/s, travel range: 100 mm) to provide pullback motion. The probe was clamped such that the Y-joint and ultrasonically transparent sheath were held stationary whilst the torque coil with the optical fibres fixed inside was mounted on the translation stage (Fig. 2(c)). This enabled the OpUS probe to be pulled back within the sheath, whilst the outer sheath remained stationary. The sheath, along with contained ultrasound probe, was inserted into the mock endoscope and submerged in a water bath for imaging, while saline was injected through the side-arm to provide ultrasound coupling. To acquire ultrasound images, the probe was translated laterally at a constant velocity within the sheath with respect to the sample surface. A-lines were acquired at a lateral spacing of 25 microns. The acquisition rate was varied to maintain the A-line spacing depending on the translation velocity; i.e. for a velocity of 100 mm/s, a repetition rate of 4 kHz was used. Each A-line comprised 4000 data points which corresponded to a total imaging depth of *ca.* 30 mm. The impact of larger A-line spacing was investigated by removing A-lines in post-processing.

#### 2.2.2 Image processing

Acquired A-lines were concatenated, followed by the application of a bandpass filter (Butterworth, 4^th^ order, 1.5−40 MHz). Subsequently, a cross-talk algorithm was applied to remove the ultrasound signals transmitted directly from the generation fibre to the reception fibre, as described previously [3]. Briefly, each scan was fitted was a general linear model of three components: a local average obtained from 40 scans, the derivative of the local average to allow for temporal offsets, and a constant term. The modelled cross-talk was then subtracted from the signals.

This was followed by the application of digital time-gain compensation [17]. Parameters $i_{max}$ and γ were empirically designated as 650 and 2 respectively. The ultrasound image was reconstructed using the k-Wave toolbox [30], using a k-space method based on the fast-fourier transform. Finally, the signal envelope was found using the absolute value of the Hilbert transform followed by a log transformation.

#### 2.2.3 Imaging targets

Two imaging targets were used for this study; a resolution phantom and *ex vivo* porcine oesophagus. The resolution phantom was used to assess the probe capabilities in terms of axial and lateral resolution and signal-to-noise ratio (SNR). It comprised a plastic frame strung with regularly spaced tungsten wires (OD: 27 µm). The phantom was mounted in a water bath and angled with respect to the OpUS catheter such that the wires were positioned at increasing depths (Fig. 2(c))).

Imaging of *ex vivo* porcine oesophageal tissue (Medmeat, UK) was carried out to investigate the clinical potential of the OpUS probe. The tissue was acquired frozen, and subsequently defrosted and stored in saline for experiments. The tissue was imaged immediately after defrosting. A 10 cm section of the oesophagus was mounted in a water bath and the mock endoscope was inserted into the lumen (Fig. 2(d)). The OpUS probe was inserted through the instrument channel of the mock endoscope such that it extended out of the distal end into the oesophageal lumen. The pullback protocol outlined in Section 2.2.1 was used to acquire tissue images, with the ultrasonically transparent sheath held stationary.

## 3. Results

### 3.1 Resolution phantom

**Fig. 3.**
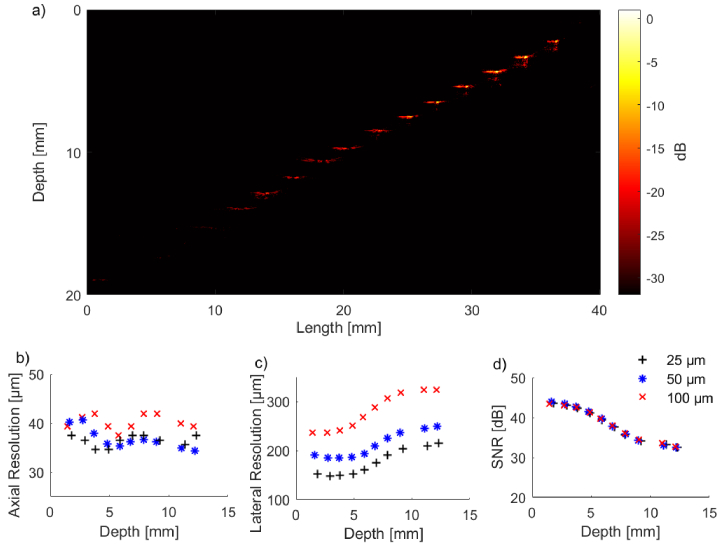
a) Reconstructed OpUS image of the tungsten wire resolution phantom acquired using a 100 mm/s fast-pullback acquisition with an A-line spacing of 25 µm. b) - d) OpUS probe performance with imaging depth for an A-line spacing of 25 µm (black cross), 50 µm (blue star) and 100 µm (red cross). b) Axial resolution. c) Lateral resolution. d) Signal to noise ratio (SNR).

**Fig. 4.**
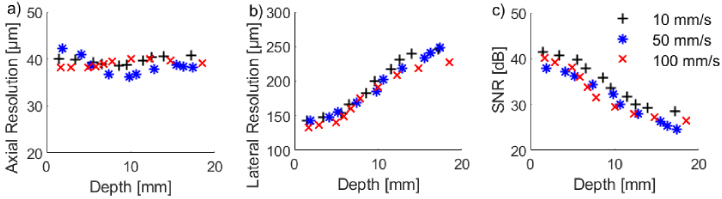
OpUS probe performance with imaging depth for an A-line spacing of 25 µm acquired using a pullback speed of 10 mm/s (black cross), 50 mm/s (blue star), and 100 mm/s (red cross). a) Axial resolution. b) Lateral resolution. c) Signal-to-noise ratio (SNR).

The tungsten wire phantom appeared in the reconstructed images as a series of point-spread function (PSF)’s which were used to measure the resolution of the imaging system (Fig. 3(a)). The full-width-at-half-maximum (FWHM) value of the resulting PSFs in the OpUS images were used to provide values for the axial and lateral resolutions. Here, all PSF’s were visible in the images acquired (Fig. 3(a)). The SNR at an A-line spacing of 25 µm decreased with increasing depth by *ca.* 1 dB/mm, from 43 dB for the closest wire ( 1.5 mm) to 32 dB for the furthest ( 12.5 mm) (Fig. 3(d)). The A-line spacing had negligible impact on the SNR values (Fig. 3(d)). Additionally, increasing imaging speed from 10 mm/s to 100 mm/s had negligible impact on the SNR (Fig. 4(c)).

For an A-line spacing of 25 µm, the lateral resolution worsened with increasing depth from 152 µm for the closest wire to 214 µm for the furthest (Fig. 3(c)). This relationship was replicated in the reconstruction at larger A-line spacing, albeit with worse overall resolution (Fig. 3(c)). The best lateral resolution for a 50 µm spacing was 190 µm, whilst for a spacing of 100 µm it increased further to 236 µm. This was also seen in the worst lateral resolutions for each spacing; at a depth of 13 mm the 50 and 100 µm spacing showed resolutions of 249 µm and 323 µm, respectively. Further, similar to the SNR, it was found that the pullback speed had a negligible impact on the lateral resolution (Fig. 4(b)).

The axial resolution was consistently better than the lateral resolution. Unlike the lateral resolution, the axial resolution was largely independent of the system parameters and remained relatively constant at 37 - 43 µm, independent of the A-line spacing, pullback speed, or target depth (Fig. 3(b), Fig. 4(a)). For example, the best axial resolution at 10 mm/s was measured to be 37 µm, while the best axial resolution at 100 mm/s was measured to be 38 µm.

### 3.2 Oesophagheal tissue imaging

**Fig. 5.**
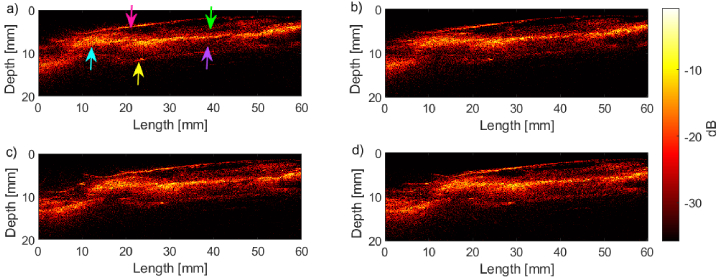
Reconstructed OpUS image of oesophageal tissue acquired using a) - b) 10 mm/s fast pullback reconstructed at an A-line spacing of a) 25 µm and b) 100 µm. c) - d) corresponding images taken using 100 mm/s fast-pullback at an A-line spacing of c) 25 µm and d) 100 µm. Right: dB scale bar. Part a) labels showing: inner edge of tissue (pink), outer edge of tissue (yellow), oesophageal mucosa (green), submucosa with oesophageal glands (blue) and muscularis propria (purple).

*Ex vivo* porcine oesophagus was imaged to investigate the clinical potential of the OpUS probe and demonstrate its capability for gastrointestinal imaging. The full thickness of the oesophageal wall was visible in the image, with both the inner (pink arrow) and outer surface (yellow arrow) appearing as distinct boundaries against the background (Fig. 5). Several distinct regions were apparent, which, on comparison with previous ultrasound images of the oesophagus, were thought to correspond to the oesopheal mucusa (green arrow), submucosa with oesophageal glands (blue arrow), and muscularis propria (purple arrow) [31]. The SNR was highest in the layer thought to correspond to the submucosa, with a value of 32 dB.

The acquisition speed was found to have minimal impact on the quality of the OpUS images from a qualitative perspective (Fig. 5(a,c)). Further, the SNR was found to decrease marginally with the increase in pullback speed, as demonstrated with the resolution phantom results. The SNR decreased with increasing depth. For the inner oesophageal surface which is shown at the shallowest depth (indicated with a pink arrow), the SNR was 28.7 dB for acquisition speeds of 10 mm/s and 28.2 dB for 100 mm/s. This similarity is duplicated in the intermediate layer, thought to be the submucosa (indicated by a blue arrow), with SNR values of 30.9 dB and 30.1 dB at 10 and 100 mm/s, respectively. In comparison, the SNR measured at the outer surface (indicated by the yellow arrow) was 19.4 dB at 100 mm/s and 16.4 dB at 10 mm/s. Additionally, despite the known reduction in lateral resolution associated with the increased A-line spacing, this had minimal impact on the qualitative outcome of the oesophageal imaging (Fig. 5(a,b)). The general oesophageal shape was preserved and subsurface details could be well visualised for all A-line spacings.

## 4. Discussion and conclusion

In this study, we demonstrate a method for sub-second acquisition of OpUS images while maintaining image resolutions and depths suitable for minimally invasive surgical applications. The device developed comprised miniaturised components which are compatible with current clinical endoscopes. The lateral ultrasound transmitter developed for the study exhibited peak-to-peak pressure in excess of 1 MPa with a corresponding −6 dB bandwidth of > 20 MHz. These are consistent with both previous high-bandwidth rGO-PDMS OpUS devices [32] and previous side-viewing transmitters [8].

Images acquired of a resolution phantom demonstrated axial resolutions < 40 µm. This is consistent with previous OpUS devices such as [23] or [32] who both recently reported axial resolutions of 50 µm, or [33] who reported an axial resolution of 35 µm. With commercial miniaturised piezoelectric probes, axial resolutions of *ca.* 100 µm have been reported with a corresponding tissue penetration of 4 - 8 mm [2,34]. As with conventional ultrasound imaging, axial resolution is typically limited by the bandwidth of both the generated ultrasound and the ultrasound receiver [35], as well as the central frequency of the ultrasound beam. Whilst the axial resolution provided here exceeds the clinical requirements, it could be improved further. This can be achieved by increasing the ultrasound reception bandwidth, or the bandwidth of the generated ultrasound pulse. The received bandwidth could be improved by decreasing the thickness of the FP cavity; however, this would create a decreased sensitivity [3]. The generated ultrasound bandwidth is dictated by both the brevity of the excitation pulse and the thickness of the composite coating [36]. The transmitted ultrasound bandwidth, and therefore the axial resolution, could likely be improved by minimising the thickness of the optical absorber or adjusting the excitation pulse duration [37]. Indeed, bandwidths of 170 MHz have been reported using a 30 picosecond laser incident on a coating of 38 µm thickness [38]. Similarly, [39] reported a bandwidth of 125 MHz using a 6 ns pulsewidth incident on a coating of 5 µm thickness. Since the brevity of the excitation pulse or the composite coating is not altered during either the change in acquisition step-size or the lateral pullback speed, it would be expected that the axial resolution remains independent of the imaging parameters such as A-line spacing or pullback speed. This was reflected in the results. The independence of the axial resolution from the A-line spacing means that less computationally intensive datasets can be acquired without sacrificing image quality. Additionally, it was found that the axial resolution was independent of the pullback speed, as expected, which suggests that the pullback speed could be increased to allow for larger imaging apertures or shorter acquisition times.

Similar to the axial resolution, good lateral resolution values was achieved in this study, with values as low as 150 µm. These values were comparable to a typical commercial ultrasound device which can achieve lateral resolutions of 200 µm depending on the bandwidth and frequency of the device [2,34,35], as well as being comparable to previously reported OpUS transducers [1,3,13]. Unlike the axial resolution, the lateral resolution showed dependence on various imaging parameters. Firstly, there was an inverse relationship demonstrated between the depth and the acquired lateral resolution. This relationship is similar to that demonstrated in previous OpUS studies [3,15]. Lateral resolution is dependent on the frequency of the ultrasound, as well as the divergence of the ultrasound beam and the accuracy of the A-line acquisition position when reconstruction methods are used. Here, we assume that the probe moved in a linear fashion with a constant velocity. However, knowledge of the probe position could be acquired through tracking or with the use of complementary technologies such as shape sensing fibres [40], which may improve reconstruction, particularly in the presence of motion. Further, since the image reconstruction here relies on the premise of whole-field insonification, a wider beam divergence will lead to a higher lateral resolution. The resolution could likely be improved by increasing the ultrasound beam divergence, for example by changing the aperture size. Reducing the aperture size would increase the beam divergence and therefore improve the lateral resolution if synthetic aperture reconstruction is used. However, this may lead to a reduction in penetration depth due to an increased fall off in pressure with depth. Alternatively, by increasing the aperture size to improve the penetration depth, the probe size could become incompatible with a typical endoscope. Even a marginal increase in size could introduce more friction during pullback within the sheath which would be detrimental to the image quality.

Secondly, there is an additional inverse relationship between the lateral resolution and the A-line spacing. Typically it is the aim to achieve the smallest possible resolution. Here, however, it would be beneficial to be able to provide real-time image reconstruction and analysis, so the compromise must be between the computational intensity of the acquired data and the resulting image quality, as mentioned previously. Using this as a baseline, the A-line spacings of 25 and 50 µm are both more than adequate for the purpose here. Finally, the last imaging parameter of consideration here is the pullback speed. This work used a rapid linear pullback for image acquisition. The use of the pullback technique resulted in drastically reduced acquisition times than those previously reported using a raster scanning approach. It appears from the results that the pullback speed has minimal impact on the resulting image quality (Fig. 4). This suggests that the probe is adequately stable during the rapid acquisition. As such, images can be acquired significantly faster without compromising on the image quality. The ability to acquire images with a short acquisition time is important for maintaining image quality in the presence of tissue motion, which will be explored in future work.

The maximum pullback velocity used in this study was 100 mm/s which enabled imaging over an 5 cm aperture in 0.5 s. The translation stage used here can reach speeds of 500 mm/s. This could be beneficial for larger imaging apertures, capable of imaging 50 cm in 1 s. The average adult human oesophagus is approximately 40 cm in length. To this end, this faster acquisition rate could acquire more pullback scans within the timespan of a regular endoscopy, thereby minimising the sampling error that is present in the acquisition and pathologic interpretation of biopsies which is currently the primary detection method of Barrett’s Oesophagus [26,27]. In this study, pullback speeds were limited to 100 mm/s. An increased pullback speed could still create an A-line spacing of 25 µm by utilising a higher repetition rate. However, this increased rate would come at the expense of a higher energy deposition rate in the coating, which may lead to heating or damage to the coating. This requires exploration in further studies if the repetition rate is to be increased further. The results here showed that even at the highest speeds with an A-line spacing of 50 µm, the resolution is still comparable to previously reported devices [3,35,41], even up to depths of 20 mm, which exceeds the largest thickness of the oesophagus. The results here show that an increased A-line spacing of 50 µm can be used without significant detraction of image quality. Therefore, using a 50 µm spacing combined with a 4000 Hz acquisition rate, the maximum applicable pullback speed is 200 mm/s, meaning the acquisition of a single pullback scan of a 100 mm aperture could be achieved in 0.5 s.

Another issue encountered at high speeds is movement in the components comprising the device. For high speeds, the transmitter and receiver stability was reduced, leading to a reduction in image quality. Whilst the motor can exceed the current maximum pullback speeds, speeds in excess of 100 mm/s create friction with the device between the two elements, which causes a reduction in the image quality. To overcome this in future studies the device fabrication method could be modified; this could include the introduction of a custom micromachined housing to hold the transmitter and receiver relative to one another. Alternatively, a single optical fibre could be used for both ultrasound reception and transmission, as demonstrated in a previous study [23]. However, the pressure generated with this device was lower than achieved here and thus may not provide the required contrast for oesophageal imaging. Further work could be carried out to optimise this device for oesophageal imaging in future studies, allowing further miniaturisation and device stability.

The *ex vivo* swine oesophageal image demonstrated clinically relevant details with the tissue layers providing differing contrast. The full thickness of the tissue was visualised and an SNR of 32 dB was achieved without the use of averaging. Further, tissue boundaries were observed and demonstrated similarities to ultrasound imaging acquired in previous studies [42,43]. The image contrast demonstrated in the wire phantom images indicates the potential for high quality images, but this was not fully realised with the images of the oesophageal tissue. This was likely caused by tissue degradation due to both storage and imaging conditions. In future work, the origin of these boundaries could be verified with tissue histology or another method of ground truth imaging. The aim of these experiments was to determine the suitability of the probe for differentiating tissue layers in a laboratory setting; as such, tissue motion was not considered. In future work, the probe and system will be developed further for *in vivo* experiments, and the effect of tissue motion on imaging will be explored. This study was limited to healthy tissue, but could be extended to include imaging of diseased tissue to assess the ability to differentiate Barrett’s Oesophagus. This might be carried out on *ex vivo* human tissue or in an animal model. The primary clinical aim of oesphageal imaging would be to ascertain the presence of high grade dysplasia commonly found with the commencement of Barretts Oesophagus, which is widely considered to be a significant precursor to oesophageal adenocarcinoma [24,25,44]. This is found in the mucosal tissue [45], which was believed to be visualised beneath the tissue surface (Fig. 5, green and blue arrows). The clear delineation of these layers, in conjunction with the high resolutions achieved, indicates the clinical potential of this device.

This work represents a significant step in the surgical translation of minimally invasive OpUS imaging. The subsecond acquisition times while maintaining competitive imaging resolutions and high imaging depths would be beneficial for many clinical applications. Additionally, the packaging of the probe in a clinically compatible catheter is a key step towards bringing OpUS through medical translation. It is expected that this device will be further developed for *in vivo* studies, with rigorous robustness testing and optimisation of handling for a preclinical environment. A further area of development is the addition of complementary imaging and therapeutic modalities. OpUS has shown promise for multi-modal devices, with previous studies including a combination with photoacoustic imaging [11] and optical ablation [10]. The presented probe could be modified to incorporate a secondary modality by using a wavelength-selective coating such as those previously presented from PDMS-AuNP [7] or PDMS-Epolight [12]. Additionally, the probe could be adapted to provide rotational ultrasound imaging similar to those demonstrated in previous studies [8]. This could be achieved by incorporating a fibre optic rotary junction to enable rotation of the ultrasound transmitter. Further, the transmitting fibre could be housed in a separate torque coil to facilitate rotation along its length. The comparable imaging parameters demonstrated by this imaging method with both other OpUS modalities such as B-mode imaging [32] or rotational imaging [8], and conventional piezoelectric ultrasound [34] is indicative of the potential of rapid pullback imaging in clinical application.

## Data Availability

The datasets generated during the current study are available from the corresponding author on reasonable request.
